# Combined Effect of Depressor Additive and Heat Treatment on the Rheological Properties of Highly Paraffinic Oils

**DOI:** 10.1021/acsomega.4c08126

**Published:** 2025-02-21

**Authors:** Laura Boranbayeva, Galina Boiko, Alexander Didukh, Raushan Sarmurzina, Zhanserik Ilmaliyev, Nina Lubchenko, Assel Kozhamzharova, Serzhan Mombekov, Saki Raheem

**Affiliations:** a Kazakh National Technical University after K.I. Satpayev, 22 Satpaev str., Almaty 050013, Kazakhstan; b Research & Development Centre of “KazTransOil” JSC, Almaty 050013, Kazakhstan; c Kazenergy Association, Astana 010000, Kazakhstan; d Institute of Metallurgy and Ore Beneficiation JSC, Almaty 050000, Kazakhstan; e School of Pharmacy, JSC “S.D. Asfendiyarov Kazakh National Medical University”, Almaty050000, Kazakhstan; f School of Life Sciences, University of Westminster, London W1W 6UW, United Kingdom

## Abstract

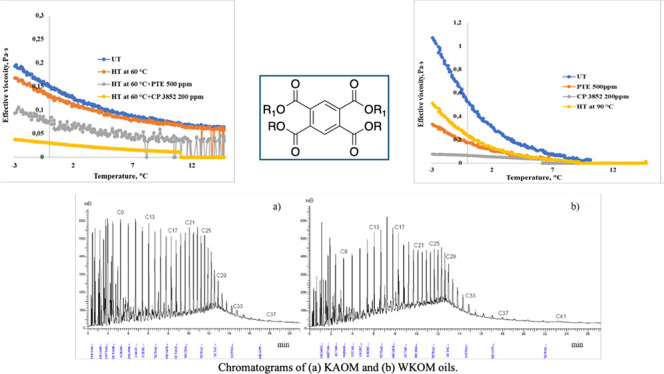

The transportation of paraffinic oils, particularly from Kazakhstan, is hindered by the formation of asphalt–resin–paraffin deposits (ARPDs), which complicate production and transport processes. While chemical treatments using inhibitors and depressants are commonly used, they are often less effective for oils with high paraffin contents and unique compositions, such as those found in Kazakhstan. This study presents a novel approach to synthesizing a depressor additive (PTE) tailored specifically for paraffinic oils, addressing the limitations of existing commercial additives. The PTE additive, derived from pyromellitic acid dianhydride (PMDA), polyoxyethylene sorbitan trioleate (Tween-85), and arachidyl alcohol (1-eicosanol), was tested on paraffinic oil blends from West Kazakhstan (WKOM) and Kumkol-Akshabulak (KAOM) under combined thermal treatment conditions at 60 and 90 °C. Rheological analyses indicated that heat treatment alone improved cold-flow properties, but these effects were transient. However, the introduction of PTE at concentrations of 500–1000 ppm produced a significant, sustained reduction in yield loss temperature (from 18 to 3 °C in WKOM and from 12 to 0 °C in KAOM) and decreased effective viscosity to 0.167 Pa s for WKOM and 0.245 Pa s for KAOM at 0 °C. Microscopic analysis confirmed that PTE alters paraffin crystallization, forming large lamellar structures that prevent network formation and maintain oil fluidity. The PTE additive demonstrated consistent effectiveness over 10 days, surpassing the stability and impact of commercial additives. These findings highlight PTE as a tailored, effective solution for enhancing cold-flow properties in high-paraffin oils.

## 1 Introduction

High-viscosity, high-liquidity oils are characterized by poor low-temperature and rheological properties,^1^ which lead to increased energy consumption during transportation and storage, necessitating specialized processing technologies.^2^ These oils typically contain solid paraffins, asphaltenes, and resins dissolved in liquid hydrocarbons, with paraffin content often exceeding 20 wt %.^2,3^ During cooling, paraffins crystallize, forming a volumetric structural lattice that encloses the liquid phase.^4,5^ The higher the molecular weight of the paraffins, the smaller their crystal size and the stronger the resulting structural lattice. As the temperature decreases, the oil transitions from a free-dispersed to a cohesive-dispersed system, where the formed structure occupies the entire volume of the oil.^6,7^

One common method to prevent crystallization during transportation is to heat the oil to temperatures of 50–60 °C.^8,9^ However, a more economically feasible approach involves using depressor additives, which interact with paraffin crystallization, inhibiting the formation of unified crystalline structure during cooling.^10−14^ This approach reduces the solidification temperature, enhances rheological properties, lowers dynamic viscosity, and reduces pressure losses from friction.

Significant research has focused on developing effective depressor reagents.^15−17^ For example, Kuman and Mahto synthesized a novel surfactant derived from sunflower oil, which functioned as a pour point depressant and allowed heavy Indian crude oil to remain fluid at temperatures as low as 1 °C. While this surfactant-based emulsification technique showed substantial viscosity reduction, its focus on a single synthesized reagent limits its applicability to other crude oils, and its long-term stability under pipeline conditions remains untested.^18^

Other studies have explored lactam-based ionic liquids (ILs) as effective depressor reagents for improving the rheological properties of heavy crude oil in high-temperature and high-pressure environments. Sakthivel and Velusmany found that lactam-based ILs, even in small concentrations, significantly reduced viscosity and yield stress, improving the flow properties. However, there are concerns regarding their long-term environmental impact due to potential IL accumulation on rock surfaces.^19^

Further research by Subramanie et al. on Malaysian crude oil investigated wax inhibitors, combining polyethylene-*co*-vinyl acetate (EVA) and poly maleic anhydride-*alt*-1-octadecene (MA) with sodium cloisite nanoclay nanoparticles. Their findings showed that nanoparticles alone reduced viscosity by 92.5% and their combination with EVA or MA further enhanced efficacy, reducing viscosity by up to 94%. However, the additives displayed inconsistent effects on shear stress, likely due to the shear-thickening behavior in the crude oil.^20^

Recently, Patel et al. explored acrylate terpolymers as pour point depressants (PPDs) for waxy crude oils. The synthesized terpolymers achieved notable reductions in pour point (up to 21 °C) and viscosity (over 75%), significantly enhancing low-temperature flow properties.^21^

In Kazakhstan, the limited availability of domestically produced depressor additives necessitates reliance on foreign reagents, which are often costly and have limited effectiveness. Developing multifunctional additives to regulate the low-temperature and rheological properties of oils with high paraffin, resin, and asphaltene contents is therefore crucial. This study aims to evaluate the effects of thermal treatment and a newly developed depressor additive (PTE) on the rheological properties of highly paraffinic oils and provides insights into potential domestic solutions for Kazakhstan’s oil industry.

## 2 Material and Methods

Pyromellitic dianhydride (dianhydride of benzene-1,2,4,5-tetracarboxylic acid) from Sigma-Aldrich was used without further purification. POES (alcoholic ester, polyoxyethylene trioleate sorbitan, Tween-85), purchased from Sigma-Aldrich, was also used without further purification. Dimethyl sulfoxide (DMSO), purchased from Sigma-Aldrich, was used without additional purification. *Para*-toluene sulfonic acid monohydrate (98.5%), purchased from Sigma-Aldrich, required no additional purification. Arachidyl alcohol (1-eicosanol C_20_H_42_O) also from Sigma-Aldrich was used without further purification.

### 2.1 The PTE Depressor Additive Was Obtained Using the Following Synthetic Methodology (Figure 1)

The synthesis of the PTE depressor additive was carried out in a three-neck round-bottom flask equipped with a magnetic stirrer, thermometer, reflux condenser, and inlet for inert gas to maintain a nitrogen atmosphere. First, 0.01 mol of pyromellitic dianhydride (PMDA) was added to the flask along with 10 mL of DMSO as the solvent. The mixture was heated to 60–70 °C with constant stirring until complete dissolution of PMDA was achieved.

Subsequently, a solution of 0.02 mol of polyoxyethylene trioleate sorbitan (Tween-85) in 10 mL of *o*-xylene was prepared and slowly introduced into the PMDA solution over approximately 10 min while maintaining the temperature between 60 and 70 °C. After the Tween-85 was added, 1.377 g of *para*-toluenesulfonic acid monohydrate (*p*-TsOH) was introduced as a catalyst. The temperature was then raised to 140 °C and held constant for 5 h.

Following this, 0.02 g of 1-eicosanol was added to the reaction flask, and the mixture was further heated and stirred at 140 °C for an additional 1 h to ensure complete reaction with the PMDA and Tween-85 components. An extra 10 mL of *o*-xylene was then added, and the temperature was maintained at 140 °C for another hour to ensure homogeneity. Upon completion of the reaction, the mixture was allowed to cool to room temperature. The resulting product was a transparent, amber-red liquid used directly as a depressor additive without further purification. The synthesized PTE was characterized by using FTIR.

**Figure 1:**
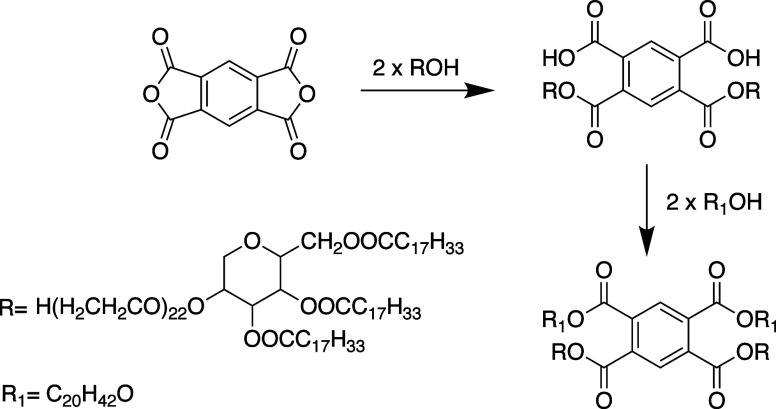
One-pot schematic synthesis of a PTE depressor additive.

### 2.2 Infrared Spectral Analysis

Infrared analysis was conducted using a Bruker spectrophotometer (FTIR) with the OMNIC software program, covering a wavenumber range of 400–4000 cm^–1^. The samples were analyzed as thin layers (films) on a potassium bromide (KBr) substrate (Bruker Optik, Germany).

**Figure 2:**
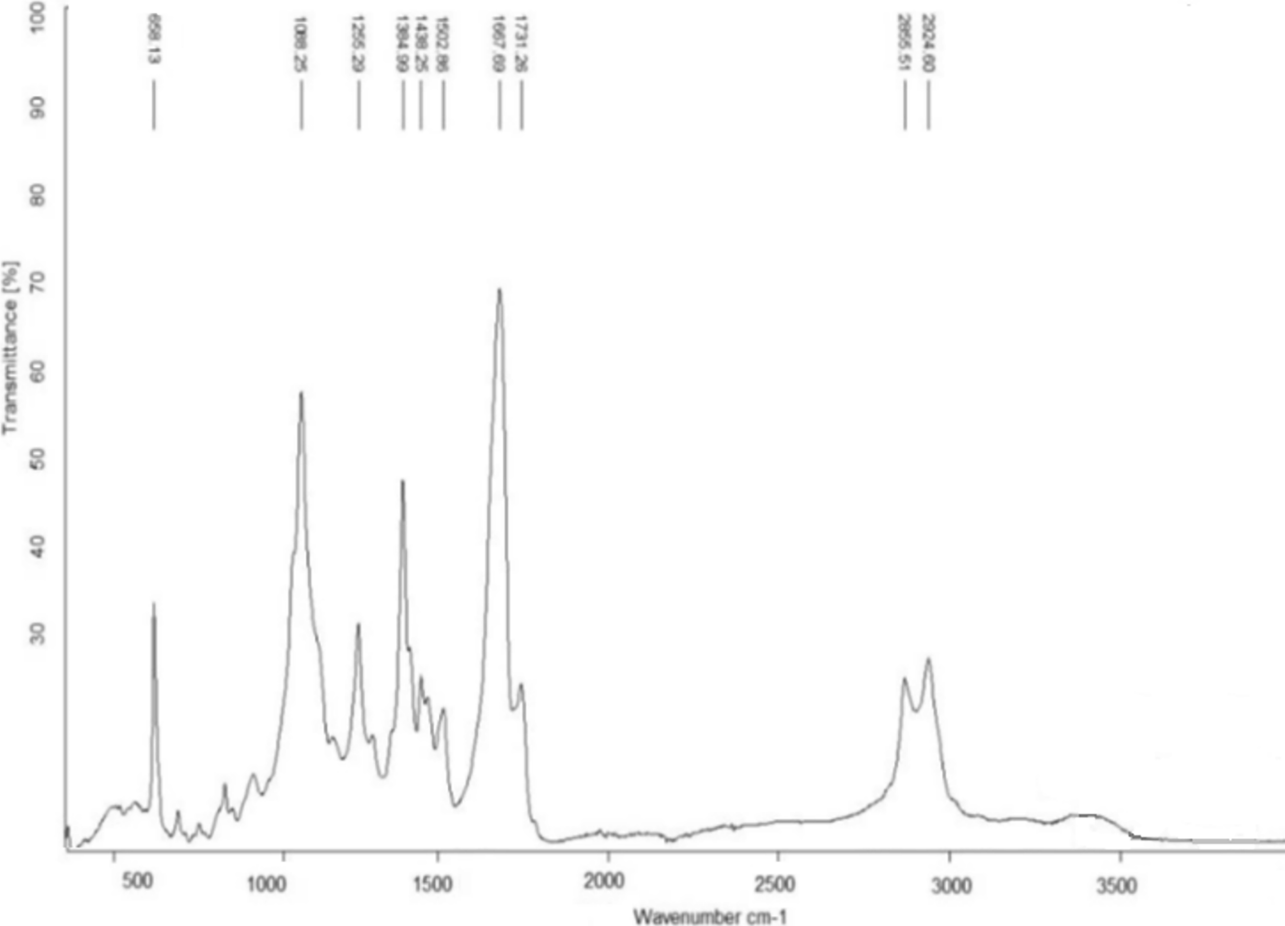
IR spectra of the depressor additive PTE.

Based on our own results (Figure 2), we conclude that the synthesized product exhibits bands at 2925 and 2850 cm^–1^, which correspond to the asymmetric and symmetric vibrations of aliphatic −CH_2_ groups and are characteristic of the Tween-85 reagent.

The absorption bands at 1722 cm^–1^ are attributed to the stretching vibrations of the carbonyl group in the ester, while the band at 1083 cm^–1^ is associated with −C–O–C– asymmetric stretching vibrations in the ester bond (O–C=O). Additionally, absorption bands at 945 cm^–1^ correspond to symmetric stretching vibrations of −C–O–C– groups in (CH_2_CH_2_O)_*n*_, a feature characteristic of the Tween-85 reagent. Characteristic vibrations of substituted benzene rings are observed in the range 1667–200 cm^–1^.

### 2.3 Oil Density

The density of oil was determined by using oil areometers according to GOST 3900 standards. A sample of the tested oil was placed in a cylinder, and a thermometer and areometer were immersed in the sample.^22^ A thermometer with an accuracy of 0.1 °C was used. The areometer reading was corrected for the meniscus effect (+0.7) and adjusted to a standard temperature of 20 °C.

### 2.4 Extraction of Paraffins, Asphaltenes, and Resins

The separation of paraffins, asphaltenes, and resins was conducted by following the GOST 11851 method. This method involves the preliminary removal of mechanical impurities and asphalt-resinous substances,^23^ followed by their extraction and adsorption. Paraffins were subsequently separated using a mixture of acetone and toluene at −20 °C. Asphaltenes were extracted from the oil (or petroleum product) using *n*-heptane and separated by filtration. The resins, dissolved in the filtrate, were adsorbed onto silica gel and then desorbed using an alcohol–toluene mixture.

### 2.5 Pour Point Temperature

The pour point temperature, or flow loss temperature, was determined by using an automatic oil pour point analyzer (OptiCPP PAC, USA) in accordance with ASTM D 5853. A metal sample temperature sensor was used,^24^ with a temperature measurement accuracy of 0.1 °C.

### 2.6 Kinematic Viscosity

Kinematic viscosity was determined according to GOST 33 using an SVM 3001 Stabinger viscometer (Anton Paar, Austria). This method outlines a procedure for the simultaneous measurement of dynamic viscosity and oil density.^25^ Kinematic viscosity is calculated by dividing the dynamic viscosity by the density, both measured at the same test temperature.

### 2.7 Effective Viscosity and Shear Stress

Effective viscosity and shear stress were measured according to GOST 1929 using a rotational viscometer (Anton Paar GmbH, Austria) with a thermostated cylindrical measuring system (C–CC39/SS, cylinder-cylinder type) and a cylindrical measuring element CC39 (sample volume = 65 cm^3^).^26^ Temperature, shear rate, and measurement frequency were controlled by using the RheoCompass software.

Measurements of apparent (or effective) viscosity and shear stress were conducted under two different conditions: (I) constant shear rate with linear temperature change: measurements were taken at a constant shear rate of 10 s^–1^ with a linearly changing temperature and (II) linear shear rate variation: this module primarily simulated severe pumping conditions, with the viscometer’s shear rate continuously increasing from 0 to 100 s^–1^ at a constant temperature.

Dynamic ultimate shear stress and yield stress were calculated by using specialized software (RHEO 2000, RheoCompass) based on the Bingham-Shvedov equation:
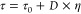
1where τ0 represents the Bingham ultimate shear stress and η is the Bingham plastic viscosity (yield strength).

### 2.8 Gas Chromatographic Analysis

Chromatographic analysis of commercial oil samples was performed using an AutoSystem LX gas chromatograph (Perkinelmer, USA) according to ASTM D2887. Sample preparation involved placing the oil sample in a preweighed container and adding the required amount of solvent.^27^ Equal amounts of each oil sample were introduced into the chromatograph.

The analysis was conducted by using a 10 m long ELITE PS 2887 capillary column with an inner diameter of 530 μm and a fixed-phase thickness of 2.65 μm. The carrier gas (Ne) flowed through the analytical column at a rate of 50 cm/min.

### 2.9 Inhibition of Asphalt–Resin–Paraffin Deposits

The inhibition of ARPDs was studied using the “cold finger” method.^28^ Paraffin deposit inhibitor evaluation unit, PR-NPH-04, was used to assess ARPD. This unit enables simultaneous testing in four vessels containing the test medium, ensuring consistent test conditions (such as mixing intensity and temperature) across all vessels.

The unit included a thermostat, an electromechanical actuator, and a test assembly. The thermostat provides uniform heating for the vessels, while the electromechanical drive ensures consistent mixing. The test assembly includes a manifold with rotameters and valves to regulate the liquid flow through U-shaped tubes.

### 2.10 Heat Treatment

For the heat treatment and additive introduction, the oil mixture under study was first heated to a target temperature of 60–90 °C. Once the target temperature was reached, the additive was directly injected into the heated oil mixture. The mixture was then thoroughly stirred to ensure even distribution of the additive throughout the oil and maintained at this temperature for 60 min to allow for optimal interaction.

Following this period, the mixture gradually cooled at a rate of 35 °C per hour. During the cooling phase, samples were collected for rheological analyses, which included the rheoviscosimetric measurement of effective viscosity, kinematic viscosity, and pour point temperature.

## 3 Results and Discussion

To study the effects of thermal treatment and the depressor additive PTE, West Kazakhstan oil mixtures (WKOMs) and Kumkol-Akshabulak oil mixtures (KAOMs), which are paraffinic high-liquidity oils, were used. Transporting these oils during colder periods presents specific challenges, as summarized in Table 1. The physicochemical and rheological parameters of those oils are detailed in Tables 1–5.

**Table 1:** Density (ρ) at 20 °C, Yield Loss Temperature (FLT), and Component Composition in WKOM and KAOM Oil Samples

no.	oil	ρ at 20 °C kg/m^3^	FLT, °C	asphaltenes, %	paraffins, %	resins, %	A/R
1.	WKOM	871.0	+15	2.725	17.15	10.50	0.259
2.	KAOM	820.1	+12	0.840	11.3	7.40	0.114

**Table 2:** Fractional Composition and Boiling Point (BHP) of WKOM and KAOM

		fraction yield, %		
oil	BHP^a^, °C	up to 200 °C	up to 300 °C	up to 350 °C
WKOM	74.4	13.6	31.8	44.6
KAOM	64.0	24.0	41.5	50.2

a Boiling point (vaporization of light fractions).

**Table 3:** Distribution of Hydrocarbons in Oils by the Number of Carbon Atoms (*n*) in the Main Chain

		hydrocarbons and paraffins, %		
no.	oil	(C_18_–C_20_)	(C_21_–C_38_)	(C_39_–C_44_)
1.	WKOM	7.89	88.98	3.13
2.	KAOM	25.39	72.42	2.19

**Table 4:** Kinematic Viscosity of Oil Mixtures at Different Temperatures

	**kinematic viscosity**, mm^2^/s				
**oil**	**20** °C	**30** °C	**40** °C	**50** °C	**60** °C
WKOM	91.16	30.20	18.06	13.64	10.95
KAOM	8.604	6.595	5.046	4.076	3.858

**Table 5:** Rheological Parameters of the Oil Mixtures

**oil sample**	*t*, °C	τ, Pa (D = 5 s^–1^)	η, Pa·s (D = 5 s^–1^)	τ, Pa (D = 10 s^–1^)	η, Pa·s (D = 10 s^–1^)	τ_0_, Pa	Y_fac_, Pa·s
WKOM	25	0.310	0.062	0.516	0.051	0	0.062
	20	0.234	0.046	0.587	0.058	0	0.088
	15	0.572	0.113	1.100	0.109	0.832	0.095
	10	2.982	0.590	4.775	0.473	3.527	0.186
KAOM	20	0.035	0.007	0.070	0.007	0	0.007
	15	0.055	0.011	0.110	0.011	0	0.011
	10	0.331	0.066	0.603	0.060	0.406	0.025
	5	2.091	0.414	3.002	0.297	2.423	0.074
	0	12.23	2.422	14.67	1.452	12.17	0.261

**Figure 3:**
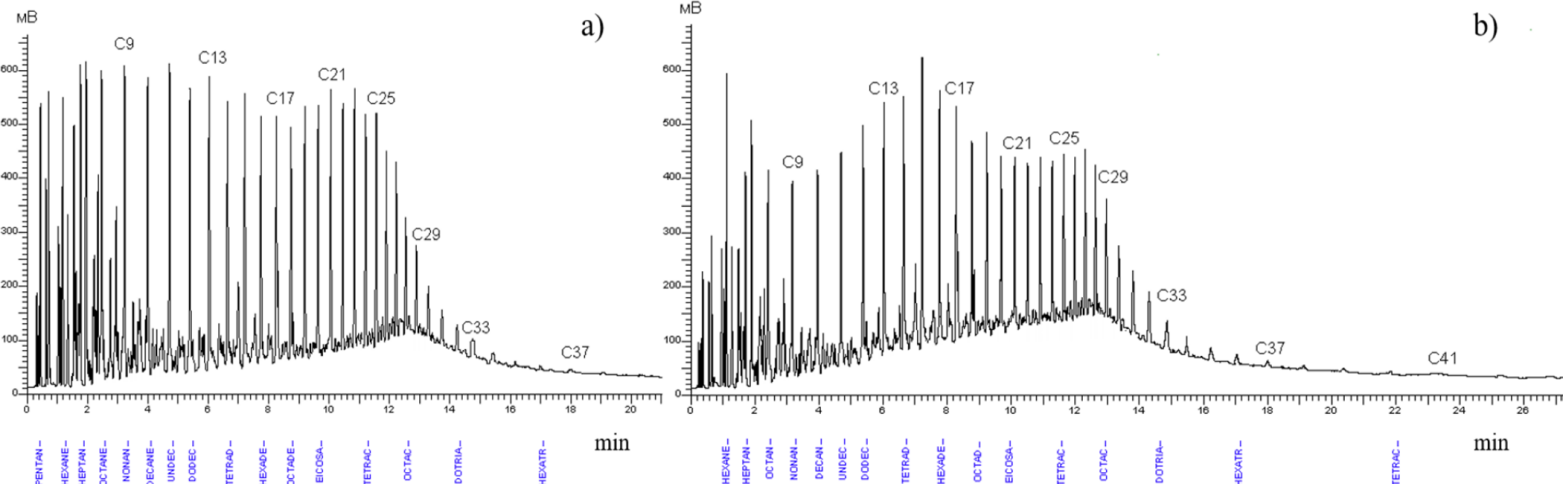
Chromatograms of (a) KAOM and (b) WKOM oils.

Table 3 compares the solid paraffins, which exhibit two maxima in all of the chromatograms of oil samples (Figure 3a,b). The separation point between these maxima occurs around the linear paraffins C_20_–C_21_, as shown in the chromatograms in Figure 3a,b. Consequently, the first group includes paraffins such as octadecane (C_18_) with a melting temperature (*T*_melt_) of 28.2°C, nonadecane (C_19_) with *T*_melt_ 32.0 °C, and eicosane (C_20_) with *T*_melt_ 36.8 °C.

The second group contains paraffins starting with genecosan (C_21_) with *T*_melt_ 40.5 °C, up to octatriacontane (C_38_) with *T*_melt_ 80.0 °C. This group forms the second maximum on the chromatograms of highly paraffinic oil samples. The third group consists of paraffins present in small amounts in all oils: nonatriacontane (C_39_) with *T*_melt_ 81.0 °C, tetracontane (C_40_) with *T*_melt_ 83.0 °C and tetratetracontane (C_44_).

The data in Table 5 clearly show that the WKOM and KAOM oil mixtures have significantly different rheological properties. The KAOM exhibits the lowest rheological parameters and a lower fluidity loss temperature (12 °C), making it more responsive to the depressor treatment. In contrast, the WKOM oil mixture shows the highest rheological parameters, with a fluidity loss temperature of 15 °C, classifying it as hardly susceptible to depressor treatment.

The observed differences in the rheological properties of these oil mixtures can be attributed to their distinct component, fractional, and hydrocarbon compositions. For example, oil viscosity correlates with resin content; higher resin content results in higher viscosity. As shown in Table 3, WKOM oil has the highest resin content, corresponding to the highest viscosity values.

The anomalous rheological characteristics, such as high yield loss temperature and a pronounced transition to a non-Newtonian state (indicated by high shear stress), are influenced by the presence of longer-chain hydrocarbons, including refractory paraffins. The higher the content of these hydrocarbons, the more pronounced the cold-flow properties, such as the yield loss temperature, paraffin crystallization temperature, limiting shear stress, and temperature at which the oil transitions to a non-Newtonian state.

According to Tables 1 and 3, the studied oil samples exhibit a high content, particularly in the WKOM oil. This aligns with the rheological properties observed in these oils and can be attributed to the significant proportion of highly paraffinic oil originating from the Mangyshlak Peninsula fields (Zhetybai, Uzen).

When the molecular-mass distribution of refractory paraffins in the oil is examined (Figure 3a,b), it is evidence that the curves for WKOM and KAOM are almost identical. This similarity, together with the component and fractional composition data, confirms a high paraffin background in both oils. In general, the composition of refractory paraffins in these oils primarily consists of paraffins with chain lengths C_19_–C_31_. A smaller portion includes paraffins with chain lengths of C_32_–C_35_, while the smallest portion comprises paraffins with chain lengths of C_36_–C_39_.

Based on gas chromatographic analysis and reference data,^27^ the following melting points are characteristic for specific groups of long-chain paraffins (Table 6).

**Table 6:** Melting Points of Long-Chain *N*-Hydrocarbons

hydrocarbons C_*n*_	C_29_	C_30_	C_31_	C_32_	C_33_	C_34_	C_35_	C_36_	C_37_	C_38_	C_39_	C_40_
*T*_melt_, °C	63.7	65.8	67.9	69.7	71.4	73.1	74.7	76.2	77.7	80.0	81.0	83.0

Given the melting points of these paraffins, it can be inferred that WKOM oil likely exhibits a heterogeneous state at 60 °C, with a significant portion of paraffin crystals remaining unmelted.

This observation aligns with the molecular-mass distribution of refractory paraffins (Figure 3a,b) and supports theoretical concepts regarding the influence of component composition (Table 3), specifically, the roles of asphaltenes and resins in structure formation within oil disperse systems.

According to modern theories,^29^ the temperature at which oil becomes saturated with paraffin is directly proportional to the mass concentration of resins and inversely proportional to the concentration of asphaltenes. Thus, the paraffin crystallization process depends on the ratio of asphaltene (A) to resinous (C) compounds in the oil. As the A/C ratio increases, the saturation temperature decreases because asphaltene associations in the oil are less stabilized due to a lower concentration of resins. This results in a reduced saturation temperature, where asphaltene associations facilitate paraffin crystallization and prevent paraffin deposition. Conversely, at low A/C values, the saturation temperature increases as asphaltenes no longer inhibit paraffin formation, allowing paraffin to precipitate freely.

Based on the analysis of the physicochemical and rheological properties of the samples, as well as data from gas chromatographic analysis of molecular-mass distribution and melting points of refractory paraffins, it is concluded that the oil heating temperature required for effective introduction of a depressor additive should not be lower than 60 °C.

**Figure 4:**
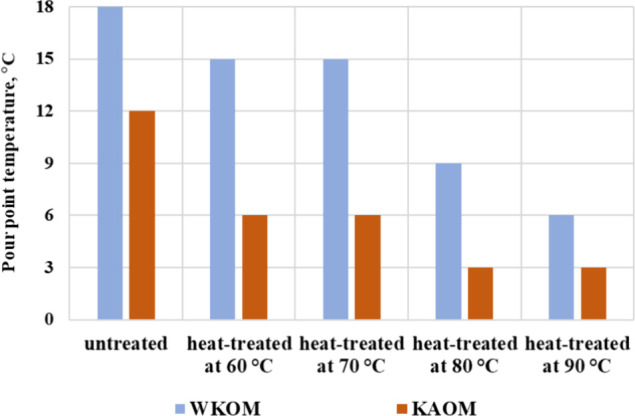
Effect of heat treatment on the yield loss temperature of the WKOM and KAOM oils.

The results show that for KAOM, heat treatment leads to a noticeable reduction in the yield loss temperature (Figure 4). The greatest reduction in yield loss temperature for Kumkol oil is 9 °C, observed after heat treatment at 70–90 °C. This effect can be attributed to the maximum paraffin content in Kumkol oil, which corresponds to chain lengths of C_27_–C_35_, requiring a melting temperature in the range of 60–65°C (Table 6).

The transition of refractory paraffins into the liquid state promotes oil homogenization, and the rheological behavior of the oil becomes similar to that of a Newtonian liquid.^30,31^ The improvement in rheological properties after thermal treatment can be explained by the fact that, as the oil cools, paraffin crystals begin to form, absorbing resinous substances.^32^ Concurrently, a structure forms that inhibits the deposition of new paraffin layers on the surface of the existing crystals. As a result, paraffin crystal growth is limited to the edges and tips.

This process leads to dendritic crystallization rather than needle-like crystallization, which would otherwise result in a three-dimensional structure within the oil. In dendritic crystallization, the paraffin in the solution forms a smaller number of larger crystals that do not create a rigid structural network.

### 3.1 Effect of Heat Treatment on Rheological Properties of WKOM and KAOM

**Figure 5:**
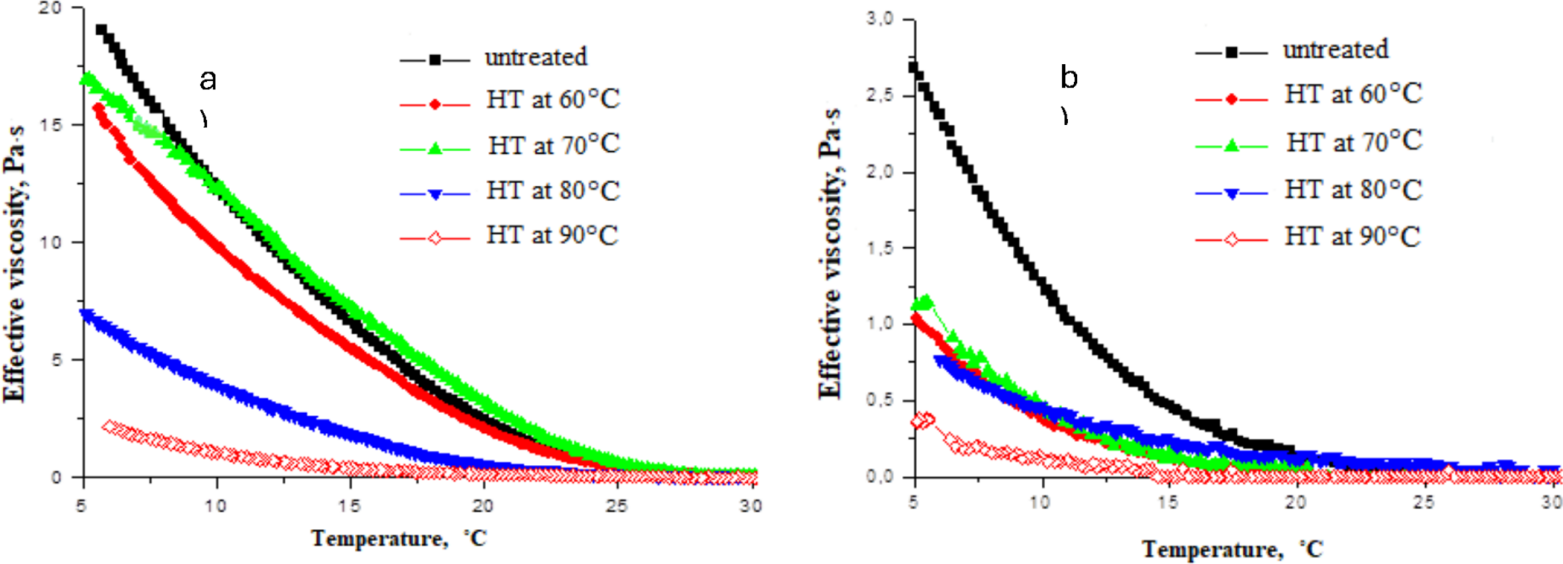
Temperature dependence of effective viscosity for untreated and heat-treated (a) WKOM and (b) KAOM at a shear rate of 10 s^–1^.

It is well known that the heat treatment of oil, depending on the heating temperature, significantly influences its rheological parameters. Heat treatment can reduce viscosity, shear stress, and yield loss temperature, as well as decrease the amount of ARPD that accumulates on the pipeline surface.^33,34^ The effects of heat treatment on the yield point and effective viscosity of the oil mixtures are listed in Figure 5a,b.

Figure 5a,b shows that increasing the oil heating temperature leads to a decrease in both the yield loss temperature and the effective viscosity. The most effective heat treatment temperature is 90 °C, where a significant reduction in effective viscosity is observed, and the decrease in yield loss temperature reaches 12 °C. WKOM is the most challenging to treat, due to its high paraffin content from the Mangyshlak Peninsula fields, mixed with resinous Buzachi oils. In contrast, the KAOM is relatively easier to treat, as it contains lighter, low-paraffin, and less complex oils from the South-Turgai fields.

The rheoviscosimetric results align well with the yield loss temperature data, showing that heat treatment up to 60 °C does not significantly alter the rheological properties of the highly paraffinic WKOM. However, heat treatment up to 90 °C results in a substantial decrease in effective viscosity. For example, untreated WKOM behaves as a Newtonian liquid at 20 °C but transitions to a viscose liquid beyond this point. This behavior is maintained even after heating the mixture to 60 °C. In contrast, for KAOM, heating to 60 °C already leads to a significant improvement in viscosity. When KAOM is heated to 90 °C, it retains Newtonian fluid characteristics down to 10 °C.

This behavior is clearly depicted in Figure 5a,b, which corresponds to the yield loss temperature data in Figure 4. Increasing the oil treatment temperature enhances its rheological properties, likely due to the roles of resins and asphaltenes, which act as natural surfactants. These components play a critical role in the paraffin crystallization process and thus influence the effectiveness of the thermal treatment for different oils.

### 3.2 Duration of the Effect of Oil Heat Treatment

**Figure 6:**
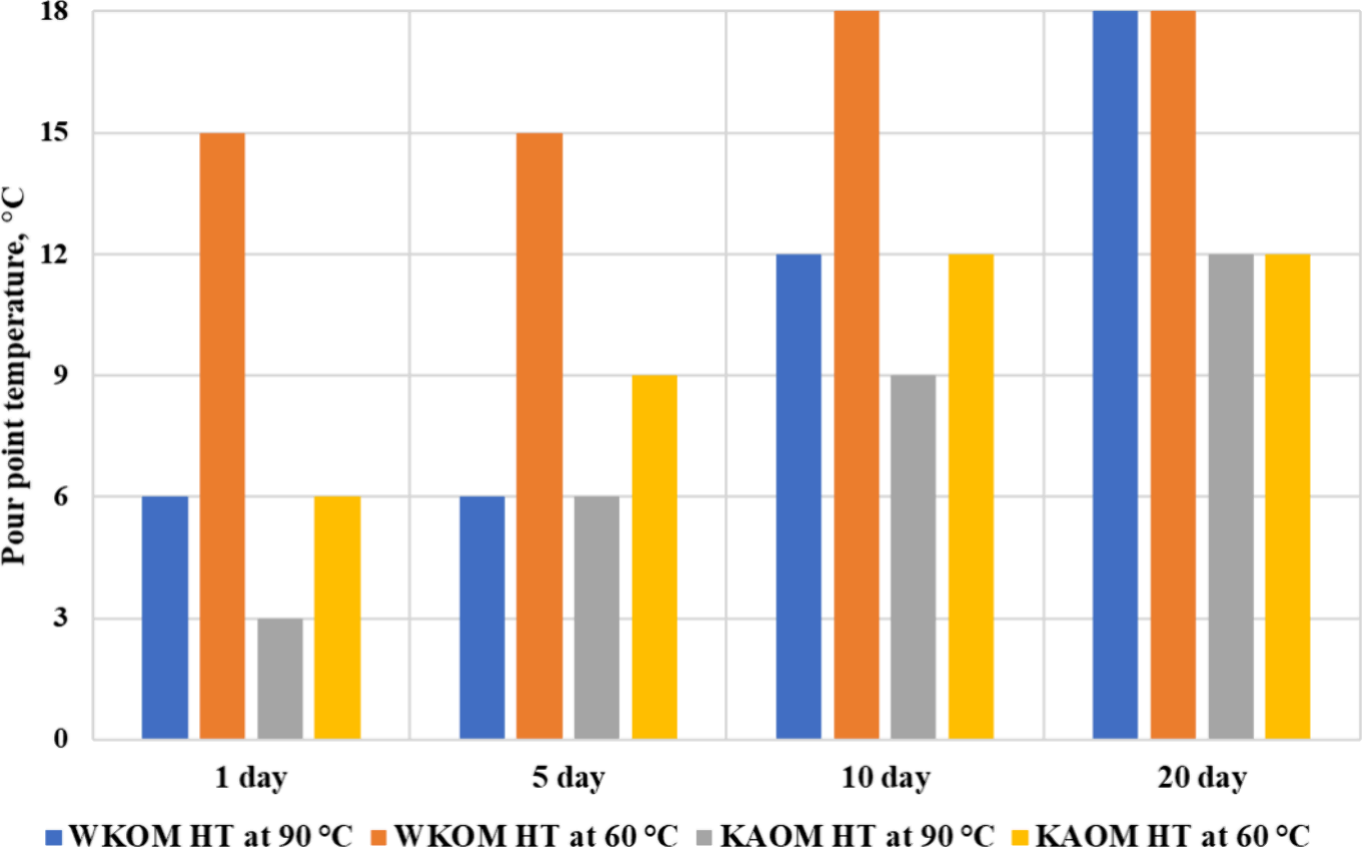
Duration of the heat treatment effect on the yield loss temperature of WKOM and KAOM.

**Figure 7:**
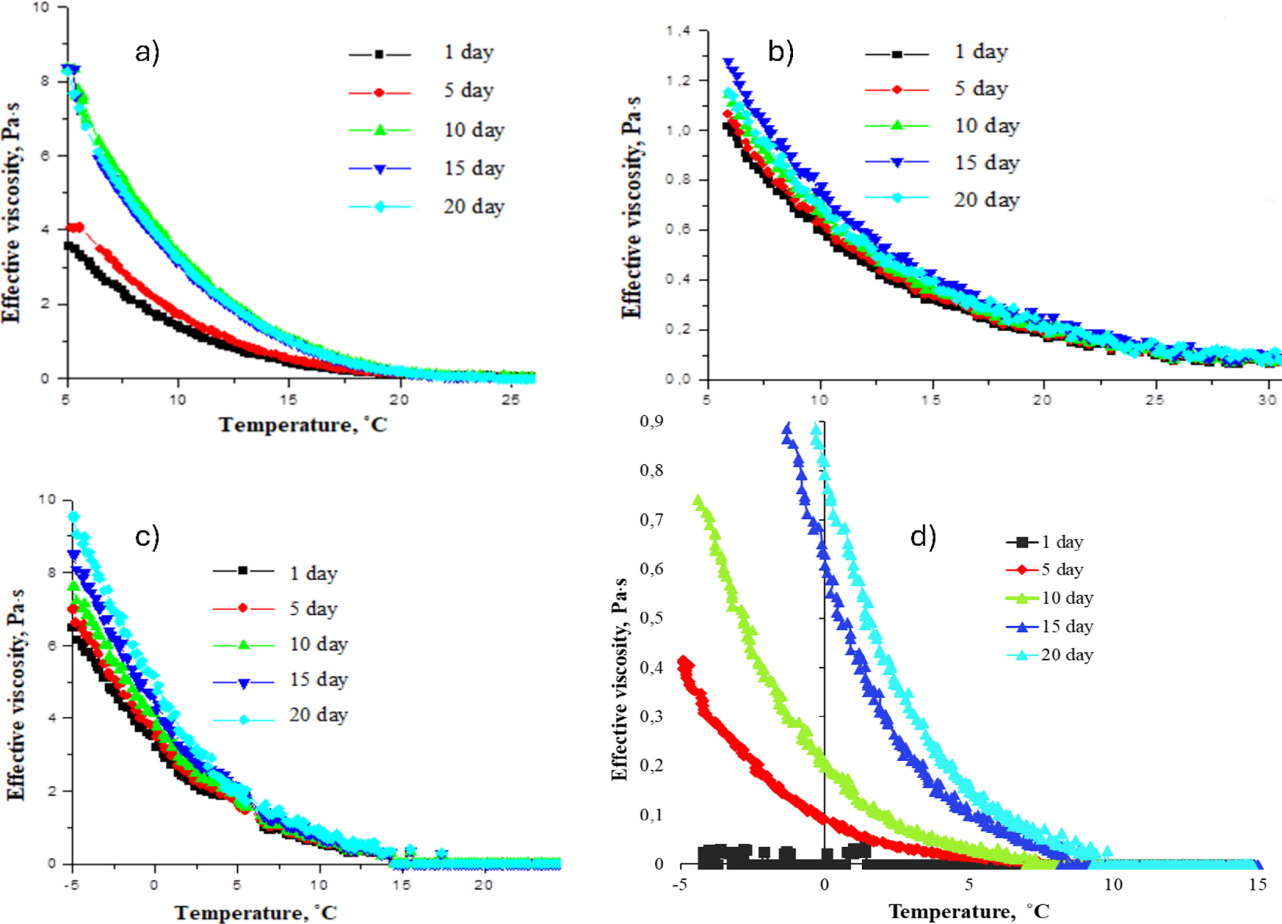
Variation in effective viscosity with temperature for heat-treated (a) WKOM and (b) KAOM at 90 °C over 20 days and for heat-treated (c) WKOM and (d) KAOM at 60 °C over 20 days. Shear rate: 10 s^–1^.

The results of studying the duration of heat treatment effects on the rheological properties of WKOM and KAOM are presented in Figure 6 and Figure 7a–d.

The data indicate that the effect of heat treatment on the oil mixtures is not stable over time. The yield loss temperature, measured on the first day after heat treatment, remains consistent for up to 5 days but begins to increase afterward. Rheological parameters, such as shear stress and apparent viscosity, also increase over time. This behavior is observed in all of the studied oil mixtures and is associated with changes in the initial structure of the oil disperse system, which forms after heating the oil to 90 °C.

The study of the duration of heat treatment effects on the rheological properties of WKOM and KAOM shows that heat treatment at 60 °C initially improves the rheological characteristics of KAOM on the 1st and 5th days. However, by the 10th day, oil fluidity begins to deteriorate. For WKOM, heat treatment at 60 °C has a minimal impact on yield loss temperature and effective viscosity.

Conversely, heat treatment at 90 °C significantly improves the cold-flow properties of both KAOM and WKOM, but this effect is maintained only up to the 5th day, after which the rheological properties begin to deteriorate.

### 3.3 Effect of Additives on Rheological Properties of Oil Mixtures

Despite the extensive research on additive effectiveness, the optimal additive choice depends on multiple factors, including the composition of the oil, the temperature at which the additive is introduced, the type and molecular weight of the polymer in the additive, its concentration, and the solvent’s polarity. Given the diversity of oils, which vary significantly in both composition and physicochemical properties, no single additive is universally effective for all oil types. Therefore, additive selection must be tailored to each oil’s characteristics, aligning the additive’s molecular structure with the components forming the crystalline phase.

Research indicates that additive molecules interact with paraffin molecules during the formation of supramolecular structures, thus inhibiting the formation of extensive crystallization centers and limiting crystal growth. This effect arises from the additive’s structure, where polar functional groups and long hydrocarbon chains allow the additive to adsorb paraffin molecules and form charged paraffin crystals.^35,36^ These interactions reduce crystal growth and encourage aggregation of smaller crystals, weakening the coagulation forces between them and thereby enhancing the stability of the oil disperse system.

Furthermore, additive efficiency may vary with temperature as optimal results are often achieved when paraffins are partially dissolved but not yet crystallized. High molecular weight polymers may also offer stronger stability around paraffin molecules, while lower molecule weight polymers are easier to disperse uniformly in the oil.

Ultimately, the selection process for an effective additive is not only a scientific challenge but also involves practical considerations such as cost-effectiveness, ease of implementation, and compatibility with the pipeline infrastructure.

To evaluate the effect of the synthesized PTE additive on the rheological parameters of oil mixtures, two temperatures were used for additive introduction: 60 °C, representing typical heat treatment conditions, and 90 °C, the recommended heat treatment temperature. Additionally, a comparative evaluation of the efficiency of the newly developed PTE additive was performed against the commercial depressor additive CP 3852 from TotalEnergies SE.

**Figure 8:**
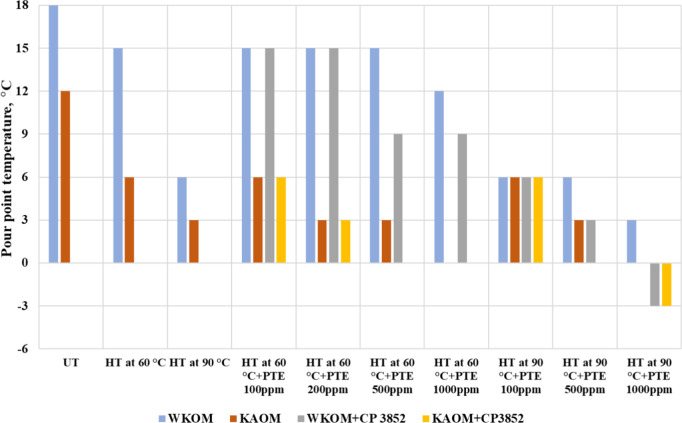
Effect of the depressor additive on the yield loss temperature of WKOM and KAOM.

**Figure 9:**
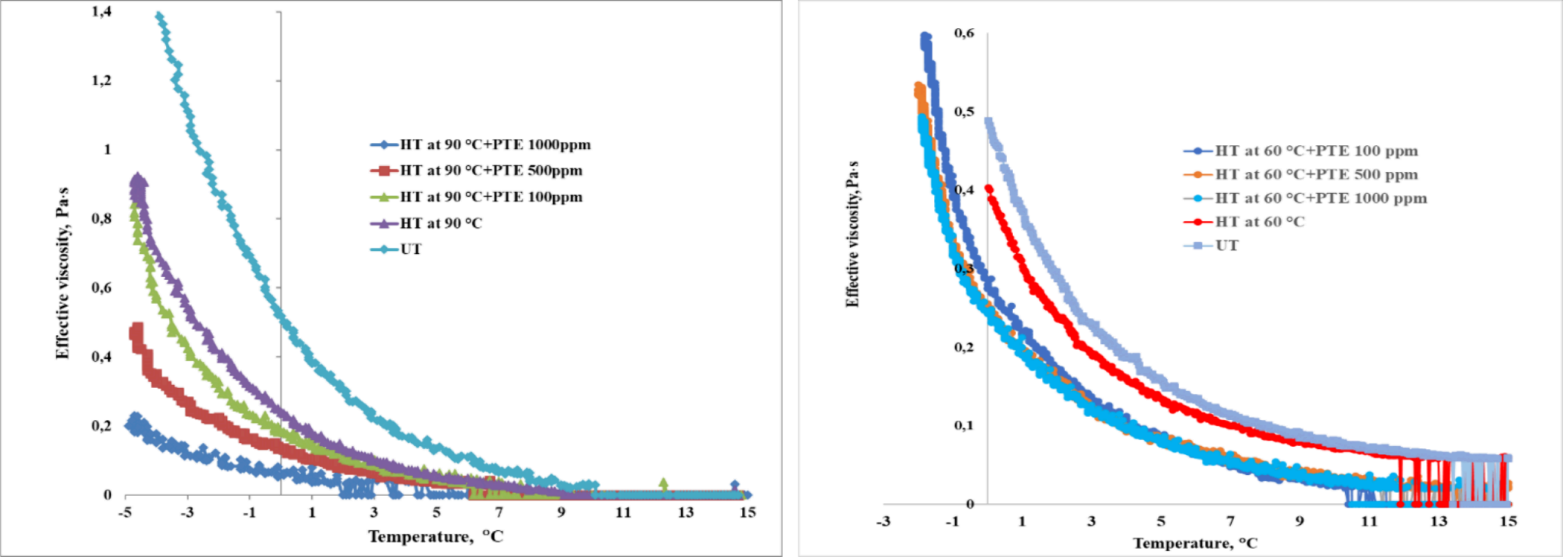
Variation in effective viscosity with temperature for (a) WKOM and (b) KAOM treated with a depressor additive. Shear rate: 10 s^–1^.

**Figure 10:**
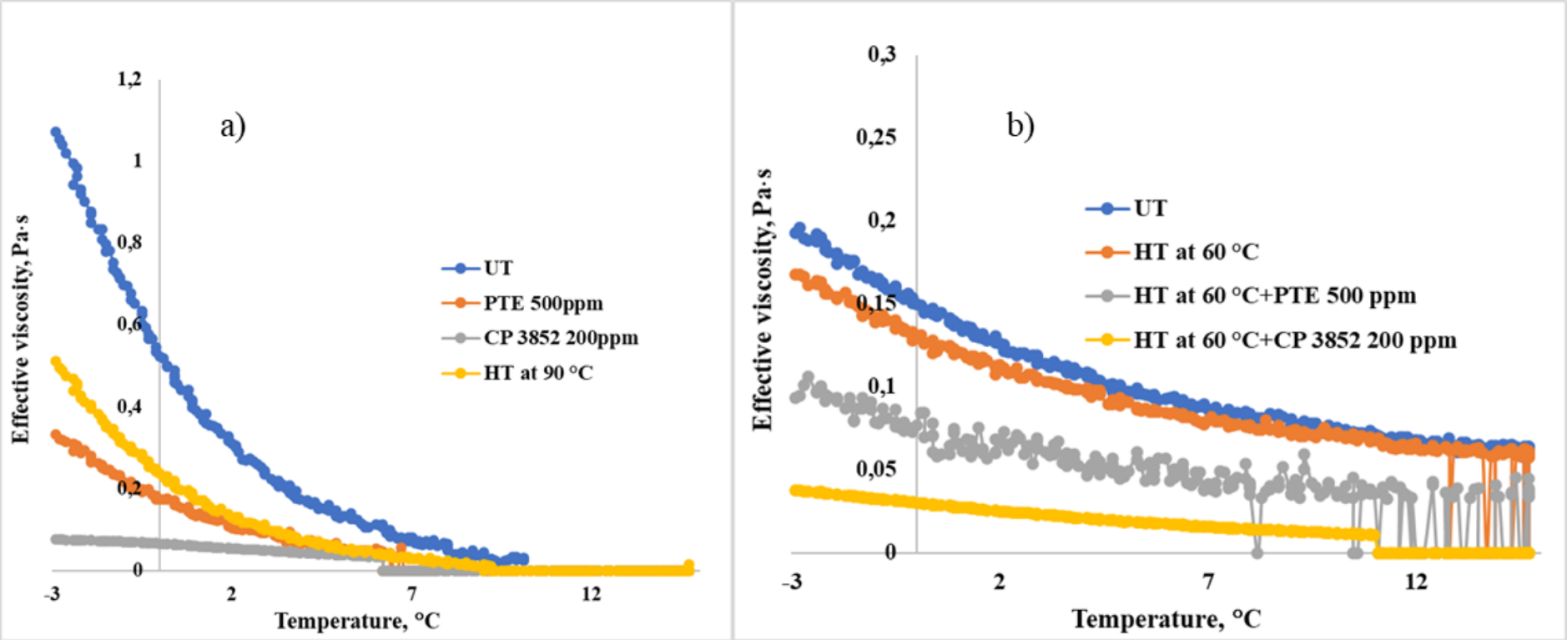
Variation in effective viscosity with temperature for (a) WKOM and (b) KAOM treated with a depressor additive. Shear rate: 10 s^–1^.

The results of the studies on the effect of PTE and CP 3852 additives on the yield loss temperature and effective viscosity of oil mixtures are shown in Figures 8, 9a, 9b, and 10a,b.

From these figures, it is evident that additives introduced into WKOM at 60 °C have a minimal effect on lowering the yield loss temperature compared to those introduced at 90 °C. For WKOM, the yield loss temperature depression after heat treatment at 60 °C is 3 °C, while treatment at 60 °C with additives yields a reduction of only 12 °C. In contrast, heat treatment at 90 °C combined with additive introduction results in a yield loss temperature reduction of 12 °C for heat treatment alone, reaching up to 15 °C with the introduction of 1000 ppm of depressor additive at 90 °C.

For the WKOM blend, the maximum yield loss temperature reduction observed is 6 °C heat treatment at 90 and 9 °C with the introduction of 1000 ppm depressor additive at 90 °C. Due to the presence of lighter paraffinic oils from the Kumkol fields, KAOMs are more easily depressurized than the WKOM.

From Figure 10a,b, a comparison of synthesized PTE additive and commercial CP 3852 additive shows that CP 3852 demonstrates the highest activity. At a concentration of 200 ppm, CP 3852 significantly improves the rheological properties of WKOM and KAOM. In comparison, the PTE additive at 500 ppm dosage is slightly less effective than CP 3852 at 200 ppm, displaying similar activity.

**Figure 11:**
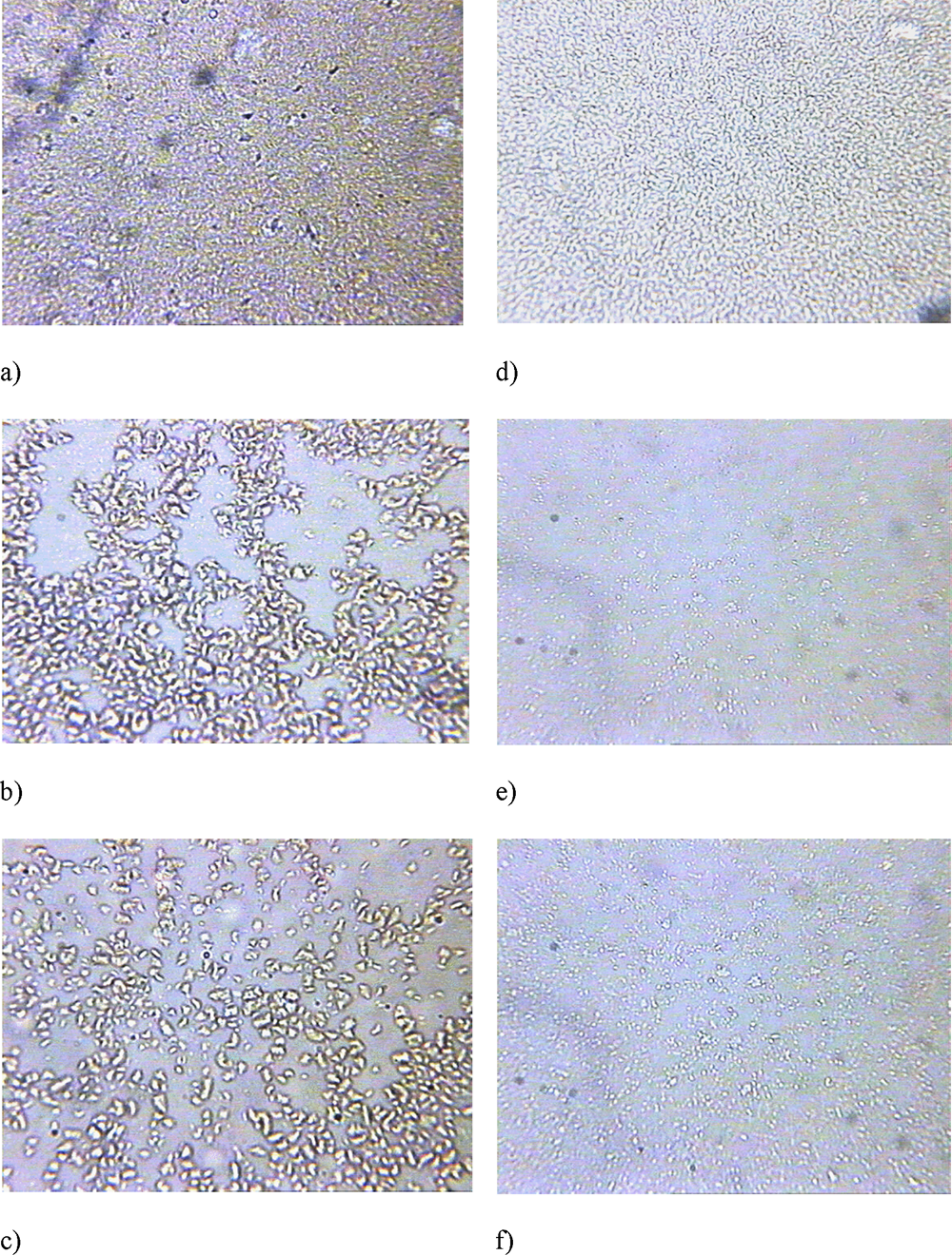
Microstructure of WKOM and KAOM oil mixtures: (a) WKOM without DA, (b) WKOM after heat treatment at 90 °C, (c) WKOM with PTE at 500 ppm, (d) KAOM without DA, (e) KAOM after heat treatment at 60 °C, (f) KAOM with PTE at 500 ppm.

The improvement in oil fluidity with the use of depressor additives is attributed to significant changes in the crystallization process within paraffinic oils, resulting in the formation of lamellar paraffin structures.^37^ This alteration is characterized by increased dispersibility, changes in crystal shape, and a reduction in the adhesiveness of paraffin crystals. These theoretical concepts are confirmed by microstructural analysis of the oil mixtures (Figure 11).

The micrographs illustrate that after heat treatment (Figure 11b,e) and additive injection (Figure 11c,f), the reticulated structure commonly seen in untreated crude oil (Figure 11a,d) does not form. Instead, large lamellar paraffin associations of various shapes are observed (Figure 11b,e,c,f). This lamellar crystal structure prevents the formation of a rigid spatial network within the oil dispersion system. At a result, the liquid dispersion medium maintains its mobility, allowing the lamellar paraffin crystals to flow more freely.

Based on Figures 8 and 9, a concentration of 500 ppm was selected as the optimal dosage for the PTE additive. Figures 8 and 9 indicate that additives introduced into the WKOM at 60 °C have minimal impact on its rheological parameters. For example, the transition temperature to a non-Newtonian state decreases slightly from 20 °C (for the oil treated at 60 °C) to 17–16 °C (for oil treated with additives at 60 °C).

Notably, the effective viscosity of WKOM treated at 60 °C, with additives, is reduced by a factor of 2 compared to oil treated at 60 °C without additives. However, more substantial reductions in rheological parameters are observed when the oil mixture undergoes heat treatment at 90 °C following additive introduction. After heating to 90 °C, the oil transitions to a non-Newtonian state at 9 °C (compared to 20 °C for untreated oil), and with additives, this transition temperature decreases to 6 °C.

### 3.4 Duration of Additive Action

**Figure 12:**
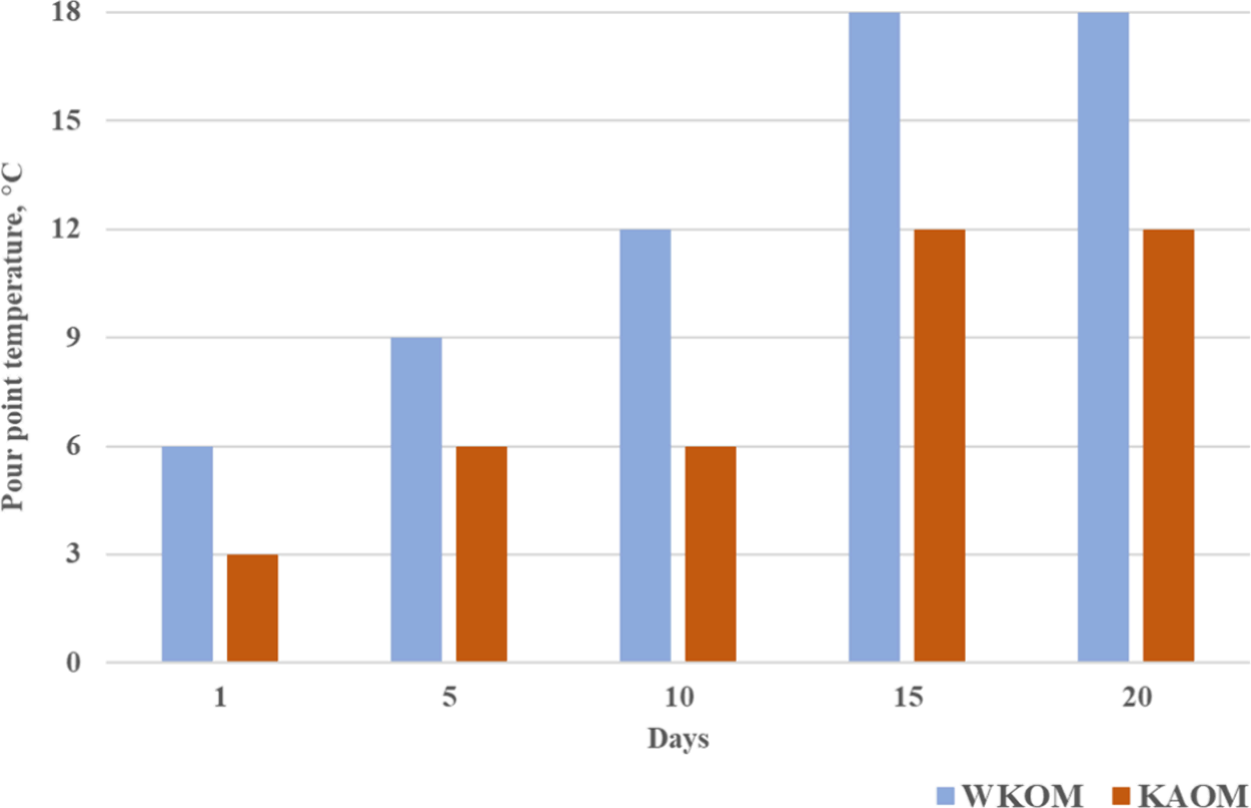
Variation in yield loss temperature for WKOM (heat-treated at 90 °C) and KAOM (heat-treated at 60 °C) with a PTE DA concentration of 500 ppm over a period of 20 days.

**Figure 13:**
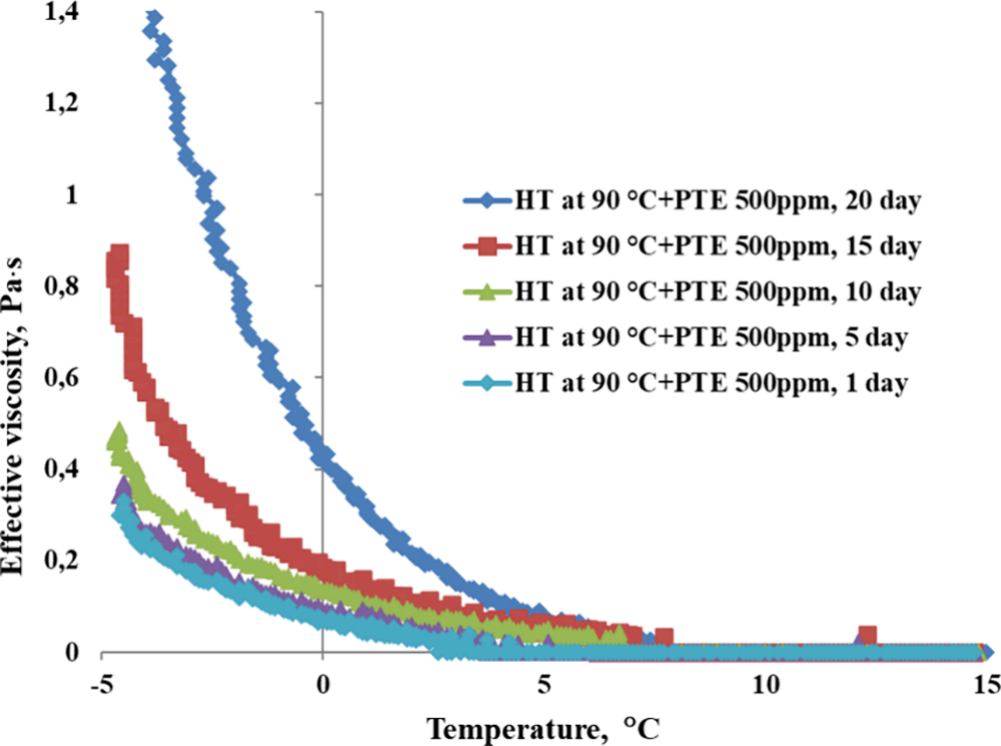
Variation in effective viscosity as a function of temperature for WKOM (heat-treated at 90 °C) with a PTE DA concentration of 500 ppm over 20 days. Shear rate: 10 s^–1^.

**Figure 14:**
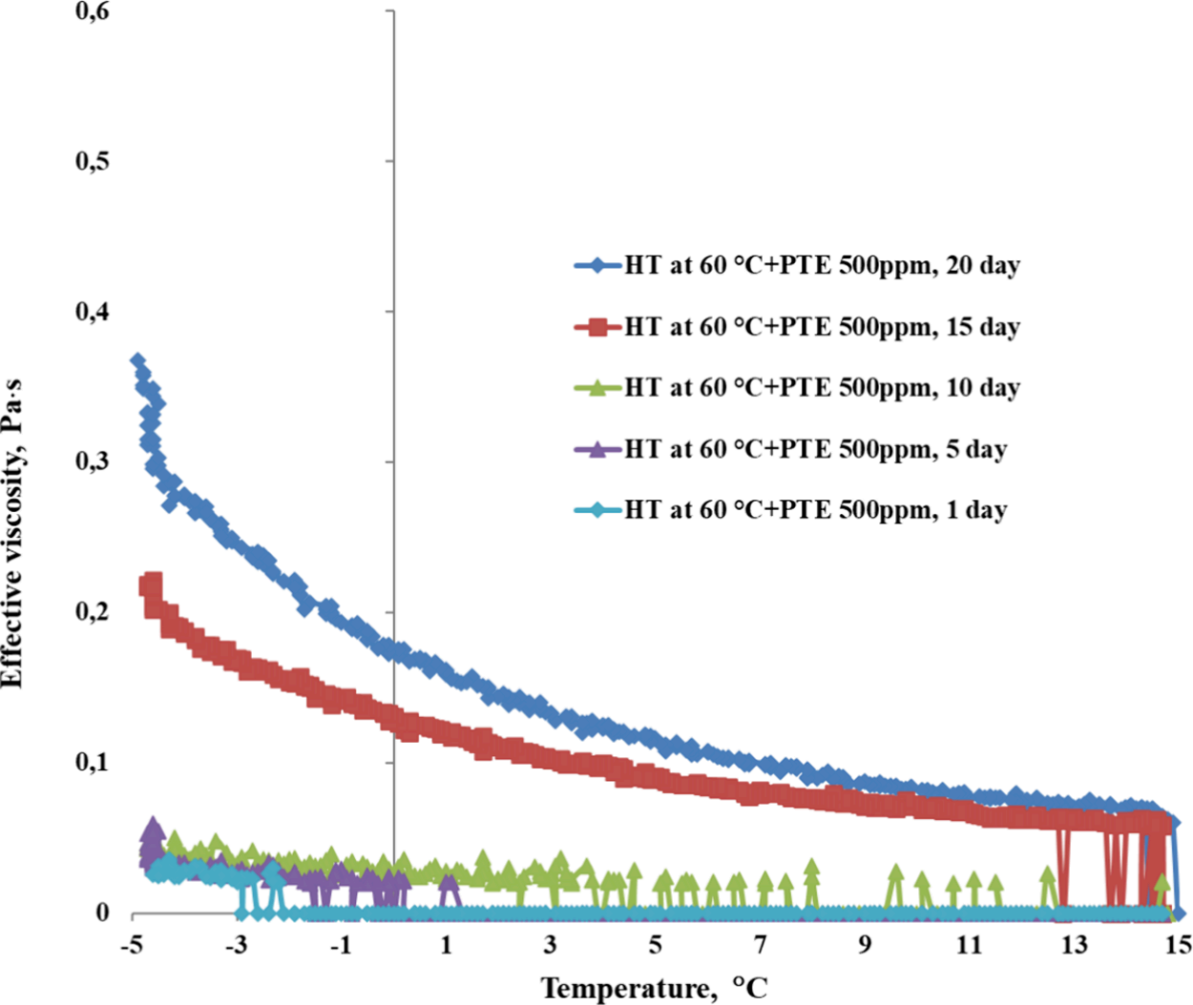
Variation in effective viscosity as a function of temperature for KAOM (heat-treated at 60 °C) with a PTE DA concentration of 500 ppm over 20 days. Shear rate: 10 s^–1^.

One of the critical factors determining the effectiveness of additives in enhancing the rheological properties of oil is the duration of their action. Figure 12 shows the stability of yield loss temperature, while Figures 13 and 14 illustrate the changes in rheological properties for WKOM and KAOM over a 20-day period.

The data indicate that the yield loss temperature and rheological parameters (shear stress and apparent viscosity) exhibit minimal changes over the first 10 days, suggesting that the PTE reagent at a concentration of 500 ppm remains stable and effective in improving the fluidity of these oil mixtures during this period.

However, after the 10-day mark, the yield loss temperature and effective viscosity begin to increase, especially for WKOM. This trend indicates a gradual reduction in the additive’s effectiveness beyond 10 days, with WKOM returning closer to its original, untreated state by day 20. In contrast, KAOM shows a more gradual increase in yield loss temperature and viscosity, retaining more of the additive’s impact over time. This suggests that the composition of KAOM may allow for a longer-lasting response to the additive treatment compared to WKOM.

The changes in effective viscosity observed in Figures 13 and 14 further illustrate these differences in stability. For WKOM (Figure 13), effective viscosity at low temperatures increases significantly between days 10 and 20, indicating that additive’s influence on cold-flow properties diminishes more quickly. KAOM (Figure 14), on the other hand, maintains a lower effective viscosity across the 20-day period, showing a more sustained improvement in flow properties.

These observations highlight that while the PTE additive is initially effective in reducing yield loss temperature and viscosity, the stability of its effects varies between oil types. WKOM may require a more frequent reapplication of the additive to maintain optimal fluidity, whereas KAOM could retain improved cold-flow properties for a longer period with less frequent treatment.

### 3.5 Effect of Additives on ARPD Inhibition

The ability of additives to inhibit wax (ARPD) formation in an oil mixture is shown in Tables 7 and 8. These tables summarize the degree of ARPD inhibition for WKOM and KAOM oil samples over a 20-day period under specified conditions (oil temperature: 40 °C, rod temperature: 15 °C, oil volume: 300 mL, experiment duration: 4 h).

**Table 7:** Degree of ARPD Inhibition in SCNS (Oil Temperature 40 °C, Rod Temperature 15 °C, Oil Volume 300 mL or 220 g, Experiment Time 4 h)

	amount of ARPD (g)/degree of ARPD inhibition (%)			
sample	1 day	5 days	10 days	20 days
untreated	6.0/0			
heat-treated 90° C	5.5/8.3			
WKOM–PTE (500 ppm)	3.3/45.0	3.4/43.3	3.8/36.6	4.5/25.0

**Table 8:** Degree of ARPD Inhibition in KAOM (Oil Temperature 40 °C, Rod Temperature 15 °C, Oil Volume 300 mL or 220 G, Experiment Time 4 H)

	amount of ARPD (g)/degree of ARPD inhibition (%)			
sample	1 day	5 days	10 days	20 days
untreated	4.0/0			
heat-treated 90° C	2.2/45.0			
KAOM - PTE (500 ppm)	1.7/57.5	1.8/55.0	1.9/52.5	2.0/50.0

Table 7 indicates that heat treatment of WKOM up to 90 °C can reduce ARPD formation by 8.3% compared to that of untreated samples. However, with the addition of the PTE depressor additive (500 ppm), ARPD inhibition for WKOM increased significantly, reaching up to 45.0% on the first day and gradually decreasing to 25.0% by day 20. This demonstrates that while the additive has a strong initial effect, its inhibition capacity diminishes over time, although it remains effective for up to 20 days.

Table 8 shows that for KAOM, heat treatment alone (up to 60 °C) was more effective in ARPD inhibition compared to that for WKOM, with a reduction of 45%. When PTE (500 ppm) was added, the inhibition level increased further, ranging from 57.5% on day 1 to 50.0% on day 20. This suggests that KAOM retains a more stable inhibition effect over time with the PTE additive compared to WKOM.

Overall, these findings suggest that while heat treatment alone can partially inhibit ARPD formation, the addition of PTE at 500 ppm substantially enhances inhibition for both oil types. Furthermore, KAOM shows a more prolonged inhibition effect, suggesting that its composition may be more conducive to sustained ARPD suppression with the additive.

## 4 Conclusions

This study examined the effects of combined oil thermal treatment at temperatures of 60 and 90 °C and the depressor additive PTE on the rheological properties of paraffinic WKOM and KAOM. The depressor additive PTE was synthesized from PMDA, polyoxyethylene sorbitan trioleate (Tween-85), and arachidyl alcohol (1-eicosanol).

Thermal treatment demonstrated that for KAOM, heating at 60 °C significantly improved oil viscosity. For WKOM, thermal treatment at 90 °C maintained Newtonian fluid properties up to a temperature of 10 °C. However, the impact of thermal treatment was transient; the yield loss temperature, measured on the first day after treatment, remained stable for up to 5 days before beginning to increase. For WKOM treated with PTE DP (500 ppm) at 90 °C and KAOM treated with PTE DP (500 ppm) at 60 °C, the additive showed a relatively stable inhibitory effect.

The addition of the PTE depressor additive in concentrations ranging from 500 to 1000 ppm lowered the yield loss temperature of WKOM from 18 to 3 °C and that of KAOM from 12 to 0 °C. Effective viscosity decreased to 0.167 Pa s for WKOM and 0.245 Pa s for KAOM at 0 °C. Notably, after the addition of the depressor additive, the yield loss temperature and rheological parameters (shear stress and effective viscosity) showed minimal change over a 10-day period, indicating the stability and effectiveness of the PTE additive (500 ppm) in improving the fluidity of oil mixtures.

While this study focused on WKOM and KAOM, future research should explore a broader range of oil mixtures to assess the additive’s general applicability. Extending the stability testing period beyond 20 days provides further insights into long-term efficacy. Additionally, an environmental impact assessment is crucial to evaluate the additive’s feasibility for broader industrial application.
